# Neuroretinal Alterations in Persistent COVID-19: A Two-Year OCT Follow-Up

**DOI:** 10.3390/jcm15145497

**Published:** 2026-07-14

**Authors:** Naiara Artiaga, Elisa Vilades, Beatriz Cordón, María Satue, Alvaro Fanlo-Zarazaga, Diego Fernández-Velasco, Elena García-Martín

**Affiliations:** 1 Department of Ophthalmology, Miguel Servet University Hospital, 50009 Zaragoza, Spain; elisavilades@hotmail.com (E.V.); beatrizcordonc@gmail.com (B.C.); mariasatue@gmail.com (M.S.); d.fer.velasco@gmail.com (D.F.-V.); egmvivax@yahoo.com (E.G.-M.); 2 Miguel Servet Ophthalmology Research Group (GIMSO), 50009 Zaragoza, Spain; 3 Aragon Health Research Institute (IIS Aragón), 50009 Zaragoza, Spain; 4 Biotech Vision Center, Quirón Ophthalmology Institute, 50012 Zaragoza, Spain; 5 Department of Surgery, Faculty of Medicine, University of Zaragoza, 50009 Zaragoza, Spain

**Keywords:** retinal nerve fibre layer (RNFL), ganglion cell layer (GCL), inner plexiform layer (IPL), long COVID, optical coherence tomography (OCT), persistent COVID-19 (PC)

## Abstract

**Background/Objectives**: To evaluate long-term neuroretinal changes in patients with persistent COVID-19 (PC) versus asymptomatic controls, using optical coherence tomography (OCT) over a two-year follow-up. **Methods**: This prospective longitudinal study enrolled 133 participants: 94 PC patients and 39 control subjects. Participants underwent comprehensive ophthalmological assessment at baseline, at 1 year (voluntary), and at 2 years. Structural retinal changes were analysed using spectral-domain OCT with the Bruch’s Membrane Opening–Minimum Rim Width and Posterior Pole protocols. Longitudinal analyses focused on within-subject change from baseline to 2 years and on between-group comparisons of these change scores, with Bonferroni correction applied within predefined families of comparisons. **Results**: The most robust corrected between-group longitudinal finding was a greater decrease in the central macular sector (C0) of the inner plexiform layer (IPL) in PC patients than in control subjects. **Conclusions**: Although these findings suggest possible subtle inner retinal involvement in PC patients, particularly in the central IPL, the results should be interpreted cautiously because of the modest control sample, control-group heterogeneity, and the exploratory nature of the sectoral OCT analyses. OCT may be useful in research settings as a complementary tool for exploring subtle neuroretinal changes in PC patients; however, its clinical utility and role as a validated biomarker remain unproven.

## 1. Introduction

Severe acute respiratory syndrome coronavirus 2 (SARS-CoV-2), the cause of coronavirus disease 2019 (COVID-19), is an enveloped, positive-sense single-stranded genomic RNA virus (+ssRNA). The first cases were reported in Wuhan, China, at the end of 2019, and the infection subsequently spread worldwide. In March 2020, the World Health Organization (WHO) declared a global pandemic [1,2]. The pandemic was characterised by successive waves of infection influenced by the emergence of viral variants, including Delta and Omicron, together with changing levels of population immunity due to infection and vaccination [2].

Persistent COVID-19 (PC), also referred to as long COVID, is defined by symptoms that persist or develop again following acute SARS-CoV-2 infection, usually after 4–12 weeks, and cannot be explained by alternative diagnoses [3]. Symptoms may include dyspnea, fatigue, arthralgia, myalgia, chest pain, dysphonia, cough, headache, anosmia, depression, anxiety, cognitive impairments, and ageusia. These occur with varying degrees of both intensity and potential impairment of quality of life [3,4,5].

The ocular manifestations of SARS-CoV-2 infection and PC are still not fully understood [6]. Several studies using optical coherence tomography (OCT) in post-COVID-19 patients have reported retinal changes with potential neurological implications [6,7]. OCT is a non-invasive imaging technique that enables high-resolution evaluation of the optic nerve and retina, which are considered anatomical extensions of the central nervous system (CNS) [7]. It can detect changes in the ganglion cell complex and retinal nerve fibre layer that may reflect neuroaxonal injury, neuroinflammation, or systemic neurodegeneration. Structural assessment of the optic nerve and retina using OCT may therefore provide an indirect window into CNS involvement [6,8].

Previous studies involving PC patients have reported inner plexiform layer (IPL) and ganglion cell layer (GCL) thinning, along with a thickening of the inner nuclear layer (INL) of the retina [6,9]. Moreover, decreased macular thickness in the superior and temporal regions, thinning of the retinal nerve fibre layer (RNFL) in the temporal segment, and generalised choroidal thinning, even six months after infection, have been reported [9].

Burgos-Blasco et al. performed a longitudinal study with one year of follow-up that confirmed these variations, highlighting a reduction in the outer inferior thickness of the GCL, RNFL and the macula. In addition, thinning was observed in the outer nasal, outer temporal and inner regions of the RNFL. At the same time, in these patients, increased thicknesses in the central region of the GCL, RNFL and macula were recorded, as well as thickening in the outer nasal segment of the GCL [10].

Overall, available evidence suggests that SARS-CoV-2 infection may be associated with structural alterations in the retina and optic nerve [11]. However, previous studies were limited by short follow-up, small sample sizes, control-group heterogeneity, and limited layer-specific longitudinal analyses. The main purpose of this study is to provide a longitudinal evaluation of retinal alterations in PC patients using OCT imaging and to explore whether regional neuroretinal changes are associated with functional status and quality of life. Since the study is observational, the findings are interpreted as associations and not as proof of causality.

## 2. Materials and Methods

### 2.1. Study Cohort

An observational, longitudinal and prospective study was conducted on a cohort of patients diagnosed with persistent COVID-19 at Miguel Servet University Hospital (HUMS), in collaboration with the Aragón Institute for Health Research (IIS-Aragón) and Neurology Department of this hospital. The study was approved by the Research Ethics Committee of the Community of Aragón (CEICA PI22/154) and conducted in accordance with current ethical regulations. All procedures complied with the principles of the Declaration of Helsinki and all participants provided signed informed consent.

Patients aged 18 years or older with a clinical diagnosis of PC according to current international guidelines were included. A previous positive PCR or antigen test documented on clinical history was required.

The control group consisted of individuals without persistent post-COVID-19 symptoms. Previous SARS-CoV-2 infection—documented by PCR or antigen testing—was registered when available. At baseline, 38 out of 39 control subjects had SARS-CoV-2 infection documented in their medical history and had recovered without persistent symptoms, whereas one subject had no previous confirmed diagnosis of SARS-CoV-2 infection. Due to asymmetry, the control group was analysed as a single group defined by the absence of persistent symptoms rather than by infection history; stratified or sensitivity analyses according to infection history would not be statistically meaningful and would not have provided reliable estimates.

Participants were required to have a best-corrected visual acuity (BCVA) of 0.4 or better on the logMAR scale, and an intraocular pressure (IOP) of 21 mmHg or lower to reduce the likelihood of including subjects with undiagnosed glaucoma or other ocular conditions potentially associated with retinal nerve fibre loss.

When both eyes fulfilled the ophthalmological inclusion criteria and provided OCT images of sufficient quality, one eye per participant was randomly selected for statistical analysis in order to avoid inter-eye correlation.

Participants were excluded if they had refractive errors equal to or greater than 5 diopters (D) of myopia or hyperopia and/or 3 D of astigmatism; retinal disease; glaucoma; ocular opacities affecting BCVA; abnormal optic discs; corneal alterations; a history of neurological disorders; major chronic illnesses, including systemic diseases potentially affecting retinal structure (such as diabetes mellitus, arterial hypertension, autoimmune disease, and cardiovascular disease); or were taking medications known to affect retinal thickness. The exclusion criteria were designed to reduce ocular and systemic confounding by excluding participants with major chronic illnesses, neurological disorders, ocular diseases, or who were taking medications known to affect retinal thickness.

The sample included individuals who were assessed on three occasions, each visit spaced one year apart (February 2023, February 2024 and February 2025). During these visits, the visual and structural function of the neuroretina of these patients was evaluated, as well as their quality of life through questionnaires.

Clinical variables collected included time since COVID-19 diagnosis, symptom type and duration of the acute phase, time elapsed since onset of PC symptoms if present, type of PC symptomatology (neurological/non-neurological), and treatment received. To evaluate quality of life, all patients were evaluated with EuroQoL 5-Dimension 3-Level (EQ-5D-3L) questionnaire; furthermore, PC patients completed the Post-COVID-19 Functional Status (PCFS) questionnaire.

All patients received a comprehensive ophthalmological examination, including logMAR BCVA using the Early Treatment Diabetic Retinopathy Study test (ETDRS) and IOP. In addition, sectoral changes in retinal thickness in the RNFL, GCL, and IPL were measured using a Spectralis OCT (Heidelberg Engineering, Heidelberg, Germany; software version 7.0.4) device to assess the potential structural impact.

For the correlation study, the Pearson coefficient was used to explore possible relationships between clinical variables (quality of life and functionality questionnaires) and structural retinal measurements. A significance threshold of *p* ≤ 0.05 was set to consider a correlation significant. Cohen’s (1988) classification was used to interpret the magnitude of the Pearson correlation coefficient, considering values of 0.1 ≤ r < 0.3 as a small (weak) effect, 0.31 ≤ r < 0.5 as a medium (moderate) effect, and r > 0.5 as a large (strong) effect [12].

### 2.2. OCT Method

Spectral-domain optical coherence tomography (SD-OCT) was performed using Spectralis. The Follow-Up function was used to match exam location to the baseline acquisition. Only scans with a signal quality score of >25 and without relevant motion artefacts, segmentation errors, or media-opacity artefacts were accepted.

Automated segmentation was reviewed by experienced examiners and corrected when necessary; scans with persistent segmentation errors or images that could not be reliably evaluated were excluded from the analysis.

**Figure 1:**
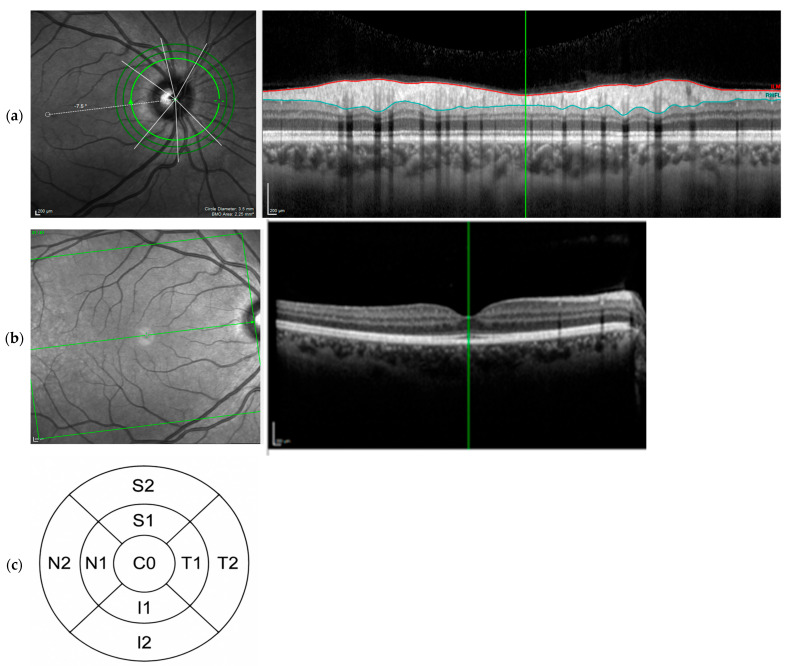
OCT acquisition protocols and sectoral segmentation used for structural analysis. (**a**) Tomographic slice and corresponding retinal location, segmented according to the Bruch’s Membrane Opening–Minimum Rim Width (BMO-MRW) protocol; (**b**) tomographic slice and corresponding retinal location, segmented according to the posterior pole protocol; (**c**) segmentation scheme of the 9 quadrants used for structural analysis, where C0 is the central macular area, S1 is the superior sector of Ring 1, S2 is the superior sector of Ring 2, T1 is the temporal sector of Ring 1, T2 is the temporal sector of Ring 2, I1 is the inferior sector of Ring 1, I2 is the inferior sector of Ring 2, N1 is the nasal sector of Ring 1, and N2 is the nasal sector of Ring 2. The coloured lines shown in panels (**a**,**b**) correspond to the automatics retinal segmentation and scan positioning, whereas the scale bars represent 200 microns (μm).

Two scanning protocols were used in image acquisition: the Bruch’s Membrane Opening–Minimum Rim Width (BMO-MRW) protocol (Figure 1a), which measures the shortest distance from the BMO to the internal limiting membrane (ILM) [13]; and the Posterior Pole protocol (Figure 1b), which evaluates the retinal layers in the macular region. The analysis method employs a macular retinal thickness map based on an 8 × 8 mm^2^ grid of small cells, each of which represents a specific area of the posterior pole [14].

Both protocols are based on the Anatomical Positioning System (APS), which ensures consistent and reproducible orientation between longitudinal examinations. Combined with the TruTrack eye-tracking system, APS ensures precise foveal localisation, even accounting for head tilt and ocular cyclotorsion [14].

The study area is segmented into 9 quadrants (Figure 1c).

### 2.3. Statistical Methods

Data was analysed using IBM SPSS Statistics, version 20.0 (SPSS Inc., Chicago, IL, USA). The normality of the variables was assessed using the Kolmogorov–Smirnov test. As most variables followed a normal distribution and each group included more than 30 participants, parametric tests were applied. Baseline between-group comparisons were performed using Student’s *t*-test or chi-square tests, as appropriate.

This interim study analysed within-group longitudinal changes in PC patients and control subjects by reporting mean ± standard deviation values at Visit 1, Visit 2, and Visit 3, together with the paired *p*-value comparing Visit 3 versus Visit 1 within each group. To compare longitudinal change between groups, intra-subject change scores were calculated for each participant as Visit 3 minus Visit 1. These change scores were then compared between PC patients and control subjects using Student’s *t*-test for independent samples.

Because the main objective was to evaluate long-term change over the two-year follow-up, this baseline-to-final visit comparison was considered the main longitudinal endpoint. Visit 2 was retained as an intermediate descriptive visit and was voluntary, particularly for control subjects, to facilitate participation and reduce loss to follow-up.

Pearson’s correlation coefficients were used for exploratory analyses between clinical variables and OCT measurements. Due to Bonferroni correction, the corrected significance threshold was *p* < 0.0056 for the nine OCT sectors by layer analysis. For the functional/clinical variables, Bonferroni correction was also applied to BCVA, IOP, and EQ-5D-3L, and only findings exceeding the relevant Bonferroni threshold were emphasised.

## 3. Results

### 3.1. Clinical and Functional Characteristics

We enrolled 133 participants, of whom 94 were patients with PC and 39 were control subjects without persistent symptoms. One participant was excluded due to insufficient OCT image quality.

In the PC group, 87.20% were female and 12.80% were male, while in the control group, 71.80% were female and 28.20% were male. The average age in the PC group was 49.6 ± 7.1 years, and in the control group, it was 52.3 ± 7.6 years, with no significant differences (*p* = 0.057). The IOP at baseline was 15.68 ± 2.61 mmHg for the PC group and 14.94 ± 2.27 mmHg for the control group (*p* = 0.107).

Functional outcomes are summarised in Table 1. In the control group, the paired Visit 1–Visit 3 comparison for EQ-5D-3L reached statistical significance (*p* = 0.007), although the magnitude of this change was small and was interpreted cautiously. In PC patients, BCVA showed a statistically significant paired change between Visit 1 and Visit 3 (*p* = 0.039). No significant paired changes were observed in BCVA in the control group or in PCFS and EQ-5D-3L in the PC group during follow-up.

PC patients showed lower scores on the EQ-5D-3L than control subjects. PCFS scores, assessed only in the PC group, indicated persistent functional impairment.

### 3.2. Cross-Sectional Spectralis OCT Findings

In the control group, no significant differences were observed in the analysed RNFL sectors. PC patients, however, exhibited significant differences in the outer nasal quadrant (N2) (*p* = 0.002) and inner superior quadrant (S1) (*p* = 0.002) (Table 2). These sectoral findings should be interpreted cautiously in the context of multiple comparisons.

In the GCL, a difference was observed in the outer superior quadrant (S2) (*p* = 0.030) in the control group (Table 3). In PC patients, differences were observed in the central macular area (C0) (*p* = 0.008), the inner nasal quadrant (N1) (*p* = 0.010), and the outer superior quadrant (S2) (*p* < 0.001).

For the IPL, the central macular area (C0) in PC patients showed a robust corrected change (*p* < 0.001). Other IPL findings, including the outer superior quadrant (S2) (*p* = 0.006) in the control group and the inner nasal quadrant (N1) in PC patients (*p* = 0.019), were interpreted as nominal or exploratory because they did not exceed the predefined Bonferroni threshold (Table 4).

### 3.3. Between-Group Changes

After analysing the changes in clinical and structural variables between Visit 1 and Visit 3 in both groups, the intra-subject change for each variable (Visit 3 minus Visit 1) was calculated. The changes observed in control subjects during follow-up were also compared with those in PC patients using Student’s *t*-test. Change scores were calculated only for participants with available paired measurements at both Visit 1 and Visit 3.

Significant differences between groups were found in IPL thickness change in the central macular area (C0) [−1.429 ± 2.458 μm in PC patients and 0.207 ± 1.177 μm in control subjects (*p* = 0.001)] (Table 5). The between-group mean difference in IPL C0 change was −1.636 μm (95% CI: −2.606 to −0.665), with a Cohen’s d of −0.79.

In addition, a significant between-group difference was observed in the change in EQ-5D-3L score [change of 0.02 ± 0.19 in PC patients and of −0.08 ± 0.16 in control subjects (*p* = 0.014)]. The between-group mean difference in EQ-5D-3L change was 0.104 (95% CI: 0.022 to 0.186), with a Cohen’s d of 0.57.

Other nominal differences, including in some GCL/IPL sectors, were interpreted cautiously and considered exploratory when they did not remain significant after correction.

Longitudinal changes were calculated using paired data from participants with available measurements at both Visit 1 and Visit 3. Therefore, mean change values may differ from the arithmetic difference between the descriptive means reported in the corresponding descriptive tables.

**Figure 2:**
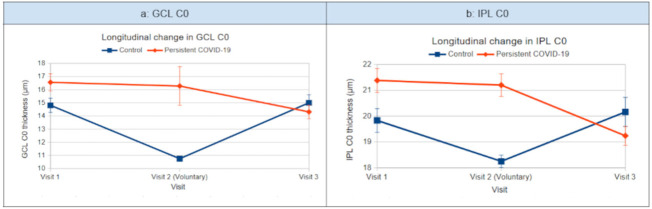
Longitudinal changes in selected central macular OCT parameters in control subjects and persistent COVID-19 (PC) patients across the three study visits. (**a**) Mean central macular ganglion cell layer thickness (GCL C0); (**b**) mean central macular inner plexiform layer thickness (IPL C0). Data are shown as mean ± standard error.

To facilitate visual interpretation of the main central macular findings, longitudinal changes in selected OCT parameters, specifically GCL C0 and IPL C0, are shown in Figure 2.

### 3.4. Correlation Analysis

**Figure 3:**
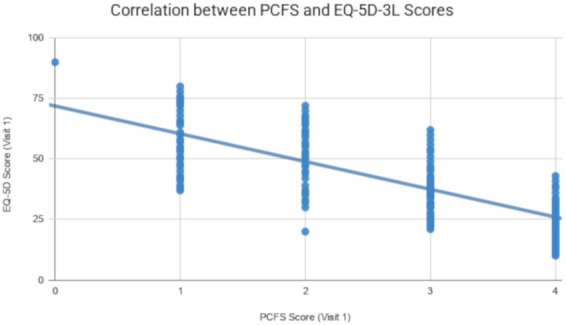
Correlation between Post-COVID-19 Functional Status (PCFS) and EuroQoL 5-Dimension 3-Level (EQ-5D-3L) questionnaire scores at initial visit in persistent COVID-19 (PC) patients. Higher PCFS scores were negatively correlated with EQ-5D-3L scores.

A strong negative correlation was identified between EQ-5D-3L and PCFS scores at Visit 1, with a coefficient of r = −0.706 (*p* < 0.001; *n* = 132). As Figure 3 shows, this negative correlation indicates that a higher perceived functional impact (higher PCFS score) corresponded to a lower reported quality of life (lower EQ-5D-3L score).

Additional low-to-moderate correlations were observed. However, because of the exploratory nature of these analyses and the number of comparisons performed, these associations were interpreted cautiously and were not considered primary findings unless clinically meaningful and statistically robust.

Regarding BCVA, a weak negative correlation with EQ-5D-3L was found at Visit 1 (r = −0.266; *p* = 0.002), suggesting an association between visual function and self-perceived quality-of-life impairment. Other statistically significant associations were identified but were not considered clinically relevant.

Correlations between longitudinal changes in study variables were also analysed. To explore potential associations between changes during follow-up and clinical development, differences between visits were calculated and correlated with clinical data.

Low-to-moderate correlations were found between changes in RNFL, GCL, and IPL thickness and clinical variables, including acute symptom duration, as well as between EQ-5D-3L and PCFS scores. A complete correlation matrix including clinical, functional, and OCT parameters is provided in Supplementary Table S1. These findings were interpreted cautiously because of the exploratory nature of the analyses and the number of comparisons performed.

Finally, weak but statistically significant correlations were also observed between changes in BCVA and clinical questionnaire scores, as well as between certain retinal layer measurements and IOP. These findings were interpreted as exploratory and were not used to support biomarker-related conclusions.

## 4. Discussion

The results of this study suggest the presence of region-specific inner retinal changes in PC patients when compared with control subjects. The most robust longitudinal between-group finding was a greater reduction in the central sector (C0) of the IPL in PC patients over the two-year follow-up. In addition, within-group longitudinal changes were observed in PC patients in the RNFL N2 and S1 sectors, in the GCL C0, N1, and S2 sectors, and in the IPL C0 and N1 sectors. In the control subjects, however, changes were more limited and were observed only in the S2 sector of both the GCL and the IPL; no significant changes were detected in RNFL sectors. These findings are consistent with previous OCT-based studies reporting retinal and choroidal structural alterations after SARS-CoV-2 infection [6,9]. However, the direction of the observed changes was not uniform, as some sectors increased whereas others decreased. Therefore, these findings should not be interpreted as evidence of generalised progressive thinning of the inner retinal layers but rather as region-specific structural alterations. Sectoral findings that did not remain significant after correction for multiple comparisons were considered exploratory and should be interpreted with caution.

Our findings suggest that the retinal alterations observed following SARS-CoV-2 infection could reflect a broader, persistent neuroinflammatory or microvascular process consistent with the neurobiological hypothesis of long COVID described by Monje and Iwasaki [5]. Since the retina is an anatomical and functional extension of the CNS, OCT may provide a non-invasive means of exploring neuroretinal changes potentially associated with systemic neuroinflammatory or neurodegenerative processes. This interpretation is also consistent with the perspective of Vujosevic et al. [8], who emphasised the value of OCT as a retinal imaging biomarker in systemic neuroinflammatory and neurodegenerative disorders. Nevertheless, the findings here should not be interpreted as evidence of a causal relationship between SARS-CoV-2 infection and retinal neurodegeneration.

With respect to the PCFS and EQ-5D-3L questionnaires, no clinically meaningful improvement was observed within the PC group during follow-up. This may reflect the chronic symptomatic state of these patients, who may not perceive substantial improvement over time. This interpretation is consistent with the study by Tak [15], which reported persistently reduced health-related quality of life, increased disability, and poorer physical and mental health among individuals with self-reported long COVID at different stages of recovery.

An intra-subject longitudinal analysis was conducted, where a greater reduction in IPL C0 thickness was observed in PC patients. Kaim et al. [16] reported a significant decrease in GCL and IPL thickness in recovered COVID-19 patients, suggesting possible inner retinal involvement after SARS-CoV-2 infection. Our findings are partially consistent with these results, although the changes observed in this study were milder and more region-specific.

Compared with studies by Sumer and Subasi [9] and Kaim et al. [16], which reported a significant thinning of the GCL in recovered patients, our results show a milder and more region-specific pattern of involvement. These differences may be attributable to methodological factors such as time since infection, severity of initial illness, or imaging technology used. Jevnikar et al. [17] previously reported that microvascular alterations tend to diminish over time, which could explain the absence of significant findings in cohorts evaluated several months after the acute phase. This time-dependency highlights the importance of conducting longitudinal studies to determine the true trajectory of post-COVID retinal changes.

A strong negative correlation was observed between PCFS and EQ-5D-3L questionnaire scores, indicating a significant association between the degree of functional disability and the subjective perception of well-being in PC patients. This finding is consistent with previous studies [18] that found severe functional impairment, deterioration in quality of life, low levels of physical activity and fatigue in PC patients. 

Low-to-moderate correlations were also observed between the changes in RNFL, GCL, and IPL measurements and clinical variables, including the duration of acute symptoms, as well as EQ-5D-3L and PCFS scores. These observations suggest that the clinical course of PC may be associated with region-specific retinal changes during follow-up. This interpretation is consistent with recent findings in PC patients, in which greater disease duration and severity were related to thinning of the GCL + IPL layers [19]. These associations should be considered exploratory and hypothesis-generating.

Analysis of retinal structural variables did not reveal strong or consistent correlations with the clinical and quality-of-life parameters evaluated. This may be due to several factors. Firstly, it is possible that the retinal changes are subtle and, therefore, have not yet manifested as evident functional alterations or as clinical perceptions captured through questionnaires. This is consistent with previous findings where subclinical structural alterations in the retina of post-COVID patients did not yield significant correlations with clinical or quality-of-life parameters [11].

Secondly, the limited duration of follow-up and sample heterogeneity may have contributed to the lack of stronger associations. Previous studies suggest that a limited follow-up may be insufficient to detect the progression or clinical manifestations of subclinical retinal changes, thus requiring longer periods to establish functional correlations [6,17]. In addition, differences in disease severity, demographics and comorbidities can hinder the detection of clear associations and account for inconsistent findings across subgroups [11,20,21].

Although no strong correlations were observed between OCT variables and clinical parameters, moderate and weak associations provide relevant insights into the relationship between retinal structure and clinical status in PC patients. The negative association between BCVA and quality of life suggests that even mild impairment in visual function can have a detectable impact on subjective well-being, as is well known [22].

Additionally, associations between specific subregions of the GCL and IPL—that jointly contribute to the transmission and processing of visual information—with functional measures were observed, supporting the idea that certain areas of the retina may be more sensitive to functional alterations [23].

The findings of this study also open up the possibility of considering OCT as a complementary follow-up tool for patients with long COVID, although its role as a validated biomarker remains to be confirmed. The incorporation of artificial intelligence algorithms, as proposed by Ortiz et al. [14], could improve the identification of subtle neuroretinal damage patterns that may remain undetected through conventional analysis. Furthermore, the combination of structural OCT and OCT-A parameters with complementary clinical metrics, including cognitive performance, fatigue, and quality of life, may enable a stronger and more comprehensive functional linkage between retinal morphological changes and the long-term manifestations of COVID-19 [18,22].

This study has several limitations. Firstly, the control sample was smaller than the PC group, reducing the statistical power of between-group comparisons and increasing the influence of control-group variability. Secondly, the control group was heterogeneous with respect to SARS-CoV-2 infection history; however, only one control subject had no previous confirmed diagnosis of SARS-CoV-2 infection at baseline, precluding stratified analyses according to infection history. Thirdly, on the one hand, major ocular, neurological, and systemic exclusion criteria were applied to reduce confounding; on the other hand, residual confounding by unmeasured or non-adjusted variables cannot be fully excluded, including tobacco use, migraine, vaccination status, number of SARS-CoV-2 infections, severity of acute episode, hospitalisation, body mass index, axial length, refractive-error distribution within the allowed range, and OCT signal quality. The availability and handling of these potential confounding variables are summarised in Supplementary Table S2. Fourthly, diagnosis based on antigen testing may have introduced misclassification because of its lower sensitivity than PCR. Fifthly, the predominance of females in the PC group and the regional nature of the cohort may limit generalisability. Finally, the study relied on sectoral OCT analyses with multiple comparisons. Moreover, although some OCT changes were statistically significant, their clinical relevance remains uncertain because of their small magnitude. Therefore, nominal findings should be interpreted cautiously and the conclusions have been restricted to the most robust corrected results.

A linear mixed-effects model would be an alternative for future analyses with longer follow-ups; however, the present analysis was based on change scores to preserve interpretability and statistical stability.

The use of the standardised Heidelberg Glaucoma Toolkit protocol enhances methodological rigour and facilitates comparison with studies on non-infectious neurodegeneration [13]. Despite the limitations described above, this study identified subtle longitudinal neuroretinal changes in patients with PC, including the central macular sector (C0) of the IPL. These findings support the possibility of subclinical neuroretinal involvement in PC, but they do not establish causality or confirm generalised progressive thinning of the inner retinal layers.

BCVA remained largely preserved during follow-up, suggesting that OCT-detectable structural changes may precede clinically evident functional impairment or may be too small to produce measurable visual changes during the current follow-up. These results highlight the value of OCT as a highly sensitive complementary tool for detecting early subtle regional neuroretinal changes and underscore the importance of regional assessments in post-COVID-19 neuro-ophthalmological studies, since while overall structural changes may be subtle, localised alterations may provide additional information.

Overall, the structural alterations observed in PC patients likely represent merely a manifestation of a broader multisystem process. However, additional evidence is required before OCT changes can be considered validated biomarkers of PC progression. Future longitudinal studies with larger and more balanced cohorts, rigorous control-group definition, OCT-A parameters, standardised image-quality thresholds, corrected statistical strategies, effect-size estimates, confidence intervals, and detailed adjustment for systemic and ocular confounders are needed to clarify the pathophysiological relevance and clinical utility of these findings.

## Figures and Tables

**Table 1:** Results of the Post-COVID-19 Functional Status (PCFS) and EuroQoL 5-Dimension 3-Level (EQ-5D-3L) questionnaires, and of the best-corrected visual acuity (BCVA) tests at Visit 1, Visit 2, and Visit 3. Visit 2 was an intermediate voluntary visit, especially for control subjects. EQ-5D-3L and BCVA were evaluated in both groups, whereas PCFS was assessed only in the persistent COVID-19 (PC) group.

Group	Functional Test	Visit 1	Visit 2	Visit 3	*p*-Value (Visit 3 vs. Visit 1)
Control	EQ-5D-3L	0.896 ± 0.226 (*n* = 39)	0.529 ± 0.179 (*n* = 4)	0.901 ± 0.160 (*n* = 33)	0.007 *
	BCVA	−0.0482 ± 0.117 (*n* = 39)	−0.050 ± 0.071 (*n* = 2)	−0.036 ± 0.074 (*n* = 33)	0.382
PC	PCFS	2.979 ± 0.854 (*n* = 94)	3.161 ± 0.626 (*n* = 56)	3.00 ± 0.875 (*n* = 48)	0.511
	EQ-5D-3L	0.503 ± 0.211 (*n* = 94)	0.506 ± 0.231 (*n* = 56)	0.506 ± 0.228 (*n* = 48)	0.402
	BCVA	−0.008 ± 0.168 (*n* = 94)	−0.017 ± 0.122 (*n* = 56)	−0.044 ± 0.068 (*n* = 48)	0.039

PCFS = Post-COVID-19 Functional Status; EQ-5D-3L = EuroQoL 5-Dimension 3-Level; BCVA = best-corrected visual acuity; PC = persistent COVID-19. PCFS was assessed only in the PC group. Descriptive values at each visit are based on all available observations at that visit. Visit 2 was voluntary and was retained for descriptive purposes only, which explains the lower number of available observations at this time point. Paired *p*-values were calculated using only participants with available data at both Visit 1 and Visit 3; therefore, paired results may differ from the arithmetic difference between the descriptive means shown for Visit 1 and Visit 3. (*) Indicates results that remained significant after Bonferroni correction (when applicable).

**Table 2:** Changes in the retinal nerve fibre layer (RNFL) in microns (μm) in control subjects and persistent COVID-19 (PC) patients. (*) Indicates the sectors that showed significant differences exceeding the Bonferroni correction.

Group	Sectors	Visit 1	Visit 2	Visit 3	*p*-Value (Visit 3 vs. Visit 1)
Control	RNFL C0	11.89 ± 1.848	10.50 ± 1.291	12.16 ± 1.969	0.621
	RNFL N1	19.47 ± 2.793	18.50 ± 1.291	19.69 ± 2.007	0.761
	RNFL N2	45.28 ± 7.988	41.75 ± 3.202	46.06 ± 7.366	0.804
	RNFL S1	23.50 ± 2.813	23.50 ± 4.041	24.62 ± 2.733	0.062
	RNFL S2	36.81 ± 4.282	34.25 ± 4.113	37.97 ± 4.239	0.939
	RNFL T1	17.00 ± 1.265	16.75 ± 1.258	17.31 ± 1.306	0.879
	RNFL T2	19.46 ± 4.773	18.00 ± 1.000	19.56 ± 4.642	0.915
	RNFL I1	24.42 ± 2.902	24.25 ± 3.202	24.03 ± 2.718	0.352
	RNFL I2	38.47 ± 4.539	35.50 ± 3.317	39.38 ± 4.156	0.943
PC	RNFL C0	12.18 ± 2.236	12.44 ± 1.870	11.49 ± 2.053	0.075
	RNFL N1	18.67 ± 2.214	18.85 ± 1.524	19.22 ± 1.403	0.056
	RNFL N2	42.55 ± 5.747	43.08 ± 5.789	43.55 ± 4.967	0.002 *
	RNFL S1	22.73 ± 3.427	22.89 ± 3.147	22.71 ± 2.082	0.002 *
	RNFL S2	35.47 ± 5.464	35.50 ± 5.050	34.53 ± 3.512	0.129
	RNFL T1	16.54 ± 1.551	16.40 ± 1.194	16.65 ± 1.422	0.118
	RNFL T2	18.47 ± 1.241	19.02 ± 4.233	18.22 ± 1.104	0.685
	RNFL I1	23.17 ± 3.095	23.37 ± 2.444	22.80 ± 2.000	0.372
	RNFL I2	37.74 ± 5.162	38.53 ± 6.543	38.35 ± 5.430	0.104

RNFL: retinal nerve fibre layer; μm: microns; PC: persistent COVID-19; C0: central macular area; S1: superior sector of Ring 1; S2: superior sector of Ring 2; T1: temporal sector of Ring 1; T2: temporal sector of Ring 2; I1: inferior sector of Ring 1; I2: inferior sector of Ring 2; N1: nasal sector of Ring 1; N2: nasal sector of Ring 2.

**Table 3:** Changes in the ganglion cell layer (GCL) in microns (μm) in control subjects and persistent COVID-19 (PC) patients. (*) Indicates the sectors that showed significant differences exceeding the Bonferroni correction.

Group	Sectors	Visit 1	Visit 2	Visit 3	*p*-Value (Visit 3 vs. Visit 1)
Control	GCL C0	14.81 ± 3.371	10.75 ± 1.258	15.00 ± 3.852	0.565
	GCL N1	49.06 ± 6.516	48.75 ± 3.096	50.06 ± 5.973	0.830
	GCL N2	41.92 ± 3.767	41.00 ± 2.944	41.31 ± 4.291	0.921
	GCL S1	52.83 ± 4.736	54.00 ± 4.082	53.13 ± 4.598	0.250
	GCL S2	36.64 ± 3.474	36.50 ± 4.123	35.81 ± 3.074	0.030
	GCL T1	47.69 ± 4.677	49.00 ± 5.354	47.22 ± 5.458	0.352
	GCL T2	37.43 ± 3.783	35.00 ± 5.196	37.38 ± 3.462	0.126
	GCL I1	52.22 ± 4.894	53.00 ± 4.082	51.72 ± 5.182	0.652
	GCL I2	34.56 ± 2.893	34.00 ± 2.944	34.34 ± 2.881	0.189
PC	GCL C0	16.56 ± 6.389	16.28 ± 14.274	14.31 ± 5.013	0.008
	GCL N1	47.51 ± 4.544	48.93 ± 4.708	50.22 ± 6.459	0.010
	GCL N2	41.73 ± 4.217	40.64 ± 4.374	41.02 ± 4.337	0.081
	GCL S1	51.72 ± 4.129	51.28 ± 5.848	51.39 ± 7.452	0.825
	GCL S2	35.68 ± 3.359	35.21 ± 3.431	34.98 ± 3.363	*p* < 0.001 *
	GCL T1	47.21 ± 5.169	46.74 ± 4.509	45.67 ± 5.764	0.301
	GCL T2	35.03 ± 4.785	35.35 ± 5.098	35.69 ± 4.705	0.110
	GCL I1	51.29 ± 5.042	51.44 ± 4.319	51.45 ± 5.657	0.877
	GCL I2	34.43 ± 4.432	33.82 ± 3.622	34.33 ± 3.716	0.222

GCL: ganglion cell layer; μm: microns; PC: persistent COVID-19; C0: central macular area; S1: superior sector of Ring 1; S2: superior sector of Ring 2; T1: temporal sector of Ring 1; T2: temporal sector of Ring 2; I1: inferior sector of Ring 1; I2: inferior sector of Ring 2; N1: nasal sector of Ring 1; N2: nasal sector of Ring 2.

**Table 4:** Changes in the inner plexiform layer (IPL) in microns (μm) in control subjects and persistent COVID-19 (PC) patients. (*) Indicates the sectors that showed significant differences exceeding the Bonferroni correction.

Group	Sectors	Visit 1	Visit 2	Visit 3	*p*-Value (Visit 3 vs. Visit 1)
Control	IPL C0	19.83 ± 2.874	18.25 ± 1.500	20.16 ± 3.522	0.352
	IPL N1	41.31 ± 3.576	40.75 ± 1.893	41.91 ± 3.863	0.562
	IPL N2	32.78 ± 2.987	32.50 ± 1.915	32.06 ± 3.047	0.204
	IPL S1	41.64 ± 3.091	43.00 ± 3.464	41.75 ± 3.069	0.261
	IPL S2	30.08 ± 2.634	29.50 ± 2.887	29.12 ± 2.472	0.006
	IPL T1	41.61 ± 3.192	42.25 ± 2.872	40.84 ± 2.919	0.318
	IPL T2	33.77 ± 2.860	31.00 ± 1.000	33.84 ± 2.438	0.169
	IPL I1	41.47 ± 3.103	43.25 ± 1.500	41.28 ± 3.255	0.152
	IPL I2	28.58 ± 2.310	27.25 ± 2.062	28.47 ± 2.110	0.556
PC	IPL C0	21.38 ± 4.462	21.20 ± 4.277	19.24 ± 3.580	*p* < 0.001 *
	IPL N1	40.39 ± 2.923	40.52 ± 5.424	41.76 ± 4.863	0.019
	IPL N2	32.34 ± 3.222	32.20 ± 3.978	31.51 ± 3.726	0.123
	IPL S1	41.12 ± 2.649	40.87 ± 5.035	40.73 ± 4.649	0.708
	IPL S2	28.97 ± 2.517	29.15 ± 3.021	28.65 ± 2.927	0.228
	IPL T1	41.11 ± 2.892	40.57 ± 4.822	40.06 ± 4.327	0.568
	IPL T2	32.16 ± 2.721	32.33 ± 3.667	32.47 ± 3.311	0.103
	IPL I1	40.70 ± 3.067	40.54 ± 5.290	40.41 ± 4.267	0.643
	IPL I2	28.13 ± 3.376	27.74 ± 4.098	28.06 ± 3.502	0.752

IPL: inner plexiform layer; μm: microns; PC: persistent COVID-19; C0: central macular area; S1: superior sector of Ring 1; S2: superior sector of Ring 2; T1: temporal sector of Ring 1; T2: temporal sector of Ring 2; I1: inferior sector of Ring 1; I2: inferior sector of Ring 2; N1: nasal sector of Ring 1; N2: nasal sector of Ring 2.

**Table 5:** Intra-subject change for each variable observed during follow-up in control subjects versus persistent COVID-19 (PC) patients. Change scores were calculated as Visit 3 minus Visit 1 using only participants with available paired data at both visits. For the main outcomes, paired data were available for 49 PC patients and 29 control subjects for IPL C0, and for 48 PC patients and 33 control subjects for EQ-5D-3L. (*) Indicates the findings that exceeded the predefined Bonferroni correction within the relevant family of comparisons.

Parameter Change (Visit 3-Visit 1)	PC	Control	Significance (*p*-Value)
BCVA	−0.035 ± 0.116	0.018 ± 0.114	0.045
IOP	2.20 ± 2.64	1.58 ± 2.48	0.283
EQ-5D-3L	0.02 ± 0.19	−0.08 ± 0.16	0.014 *
RNFL C0	−0.306 ± 1.176	0.103 ± 1.113	0.134
RNFL N1	0.674 ± 2.41	−0.138 ± 2.416	0.155
RNFL N2	2.102 ± 4.412	−0.345 ± 7.408	0.071
RNFL S1	1.082 ± 2.271	0.793 ± 2.194	0.585
RNFL S2	0.71 ± 3.234	0.035 ± 2.398	0.329
RNFL T1	0.367 ± 1.616	−0.035 ± 1.21	0.250
RNFL T2	0.063 ± 1.060	−0.138 ± 6.880	0.843
RNFL I1	−0.286 ± 2.217	−0.483 ± 2.747	0.730
RNFL I2	0.918 ± 3.883	−0.035 ± 2.570	0.243
GCL C0	−1.245 ± 3.172	0.138 ± 1.274	0.028
GCL N1	2.51 ± 6.558	−0.069 ± 1.71	0.042
GCL N2	−0.796 ± 3.122	−0.035 ± 1.861	0.237
GCL S1	0.225 ± 7.084	0.379 ± 1.741	0.908
GCL S2	−1.122 ± 1.856	−0.828 ± 1.947	0.507
GCL T1	−0.837 ± 5.599	0.414 ± 2.353	0.257
GCL T2	0.875 ± 3.722	−0.552 ± 1.882	0.059
GCL I1	−0.102 ± 4.575	−0.172 ± 2.037	0.938
GCL I2	0.041 ± 2.958	−0.310 ± 1.892	0.568
IPL C0	−1.429 ± 2.458	0.207 ± 1.177	*p* < 0.001 *
IPL N1	1.429 ± 4.108	0.241 ± 2.214	0.155
IPL N2	−0.674 ± 3.003	−0.310 ± 1.285	0.539
IPL S1	0.225 ± 4.175	0.379 ± 1.781	0.850
IPL S2	−0.388 ± 2.225	−0.862 ± 1.552	0.315
IPL T1	−0.347 ± 4.221	−0.276 ± 1.461	0.931
IPL T2	0.438 ± 1.821	−0.345 ± 1.317	0.047
IPL I1	−0.225 ± 3.368	0.345 ± 1.261	0.386
IPL I2	0.143 ± 3.149	−0.172 ± 1.56	0.616

PC: persistent COVID-19.

## Data Availability

The data presented in this study are available on request from the corresponding author due to privacy and ethical restrictions related to patient data.

## Supplementary Materials

The following supporting information can be downloaded at: https://www.mdpi.com/article/10.3390/jcm15145497/s1, Table S1: Correlation analysis between clinical, functional and OCT parameters; Table S2: Availability and handling of potential cofounding variables.

## Abbreviations

The following abbreviations are used in this manuscript:

APS	Anatomical Positioning System
BMO-MRW	Bruch’s Membrane Opening–Minimum Rim Width
CEICA	Ethics Committee of the Community of Aragón
CNS	Central Nervous System
COVID-19	Coronavirus Disease 2019
D	Diopters
EQ-5D-3L	EuroQoL 5-Dimension 3-Level
ETDRS	Early Treatment Diabetic Retinopathy Study
GCL	Ganglion Cell Layer
IIS-Aragón	Aragón Institute for Health Research
ILM	Internal Limiting Membrane
INL	Inner Nuclear Layer
IOP	Intraocular Pressure
IPL	Inner Plexiform Layer
OCT	Optical Coherence Tomography
ONH	Optic Nerve Head
PC	Persistent COVID-19
PCFS	Post-COVID Functional Status
RNFL	Retinal Nerve Fibre Layer
SARS-CoV-2	Severe Acute Respiratory Syndrome Coronavirus 2
SD-OCT	Spectral-Domain Optical Coherence Tomography
BCVA	Best-corrected Visual Acuity
WHO	World Health Organization
+ssRNA	Positive-Sense Single-Stranded Ribonucleic Acid
