# Supplementary material for: Neuroretinal Alterations in Persistent COVID-19: A Two-Year OCT Follow-Up
Source: J Clin Med. 2026 Jul 14;15(14):5497. doi: 10.3390/jcm15145497 (PMC13413350; doi:10.3390/jcm15145497)
Supplement: Supplementary file 1 [file jcm-15-05497-s001.zip › jcm-4364040-supplementary.pdf]

Supplementary Materials

Neuroretinal Alterations in Persistent COVID-19: A Two-Year OCT Follow-Up

Supplementary Table s1. Correlation analysis between clinical, functional and OCT parameters.

		DAYS_1	DAYS_2	DAYS_3	VA_1	VA_2	VA_3	IOP_1	IOP_2	IOP_3	EQ-5D-3L_1	EQ-5D-3L_2	EQ-5D-3L_3	PCFS_1	PCFS_2	PCFS_3
DAYS_1	Pearson Correlation	1	,964**	,889**	-,013	-,130	-,416**	,194	,326*	-,383**	,112	,292*	,063	-,103	-,365**	-,222
	Sig. (2-tailed)		,000	,000	,903	,342	,003	,064	,015	,006	,285	,031	,673	,325	,006	,130
DAYS_2	Pearson Correlation	,964**	1	,678**	-,179	-,073	-,471*	,053	,251	-,278	,345**	,306*	-,101	-,305*	-,271*	-,417
	Sig. (2-tailed)	,000		,001	,180	,595	,027	,695	,065	,189	,008	,022	,655	,020	,043	,054
DAYS_3	Pearson Correlation	,889**	,678**	1	-,374**	-,523*	-,421**	-,250	,355	-,271	,138	-,216	,000	-,119	-,363	-,130
	Sig. (2-tailed)	,000	,001		,009	,018	,003	,086	,125	,063	,349	,361	,998	,421	,116	,377
VA_1	Pearson Correlation	-,013	-,179	-,374**	1	,535**	,373**	,115	,054	,131	-,266**	-,168	-,093	,124	,196	,283
	Sig. (2-tailed)	,903	,180	,009		,000	,001	,197	,691	,236	,002	,199	,409	,232	,147	,051

VA_2	Pearson Correlation	-,130	-,073	-,523*	,535**	1	,595**	,073	-,026	,052	-,267*	-,378**	,201	,137	,460**	,244
	Sig. (2-tailed)	,342	,595	,018	,000		,006	,589	,851	,818	,044	,004	,395	,318	,000	,299
VA_3	Pearson Correlation	-,416**	-,471*	-,421**	,373**	,595**	1	,239*	-,169	,048	-,075	,165	-,023	,193	,214	,178
	Sig. (2-tailed)	,003	,027	,003	,001	,006		,032	,477	,670	,507	,486	,837	,188	,365	,225
IOP_1	Pearson Correlation	,194	,053	-,250	,115	,073	,239*	1	,691**	,280*	-,197*	,014	-,095	,008	,104	,193
	Sig. (2-tailed)	,064	,695	,086	,197	,589	,032		,000	,010	,026	,918	,398	,938	,449	,188
IOP_2	Pearson Correlation	,326*	,251	,355	,054	-,026	-,169	,691**	1	,129	-,188	-,103	,185	,061	,085	-,283
	Sig. (2-tailed)	,015	,065	,125	,691	,851	,477	,000		,566	,162	,444	,436	,661	,538	,227
IOP_3	Pearson Correlation	-,383**	-,278	-,271	,131	,052	,048	,280*	,129	1	-,255*	-,181	-,299**	,292*	,295	,182
	Sig. (2-tailed)	,006	,189	,063	,236	,818	,670	,010	,566		,020	,420	,007	,040	,182	,215
EQ-5D-3L_1	Pearson Correlation	,112	,345**	,138	-,266**	-,267*	-,075	-,197*	-,188	-,255*	1	,817**	,783**	-,706**	-,589**	-,474**

	Sig. (2-tailed)	,285	,008	,349	,002	,044	,507	,026	,162	,020		,000	,000	,000	,000	,001
EQ-5D-3L_2	Pearson Correlation	,292*	,306*	-,216	-,168	-,378**	,165	,014	-,103	-,181	,817**	1	,685**	-,676**	-,727**	-,665**
	Sig. (2-tailed)	,031	,022	,361	,199	,004	,486	,918	,444	,420	,000		,001	,000	,000	,001
EQ-5D-3L_3	Pearson Correlation	,063	-,101	,000	-,093	,201	-,023	-,095	,185	-,299**	,783**	,685**	1	-,541**	-,085	-,490**
	Sig. (2-tailed)	,673	,655	,998	,409	,395	,837	,398	,436	,007	,000	,001		,000	,721	,000
PCFS_1	Pearson Correlation	-,103	-,305*	-,119	,124	,137	,193	,008	,061	,292*	-,706**	-,676**	-,541**	1	,685**	,444**
	Sig. (2-tailed)	,325	,020	,421	,232	,318	,188	,938	,661	,040	,000	,000	,000		,000	,002
PCFS_2	Pearson Correlation	-,365**	-,271*	-,363	,196	,460**	,214	,104	,085	,295	-,589**	-,727**	-,085	,685**	1	,408
	Sig. (2-tailed)	,006	,043	,116	,147	,000	,365	,449	,538	,182	,000	,000	,721	,000		,074
PCFS_3	Pearson Correlation	-,222	-,417	-,130	,283	,244	,178	,193	-,283	,182	-,474**	-,665**	-,490**	,444**	,408	1
	Sig. (2-tailed)	,130	,054	,377	,051	,299	,225	,188	,227	,215	,001	,001	,000	,002	,074	
RNFL_Mean	Pearson Correlation	,151	,087	,033	,112	-,125	-,040	-,194*	-,165	-,156	-,027	,141	,097	-,004	-,216	,310*

_C0_µm_1	Sig. (2-tailed)	,151	,518	,826	,206	,358	,725	,031	,223	,166	,761	,287	,400	,973	,114	,032
RNFL_Mean_N1_[µm]_1	Pearson Correlation	-,143	-,103	-,141	,200*	,083	-,037	-,021	,205	,075	,164	-,012	,103	-,045	,113	,077
	Sig. (2-tailed)	,175	,447	,340	,023	,544	,750	,821	,130	,510	,064	,928	,369	,667	,410	,605
RNFL_Mean_N2_[µm]_1	Pearson Correlation	-,010	,024	-,008	,072	-,092	,082	,139	,214	,147	,272**	,109	,129	-,108	,040	,055
	Sig. (2-tailed)	,923	,860	,958	,419	,498	,476	,124	,113	,193	,002	,410	,259	,305	,771	,711
RNFL_"Mean_S1_[µm]_1	Pearson Correlation	,051	,229	,277	-,075	-,214	,035	,106	,336*	-,004	,185*	,030	,354**	-,032	,010	-,253
	Sig. (2-tailed)	,626	,086	,056	,401	,113	,762	,245	,011	,970	,036	,822	,001	,764	,941	,083
RNFL_"Mean_S2_[µm]_1	Pearson Correlation	-,047	,151	,063	-,132	-,167	-,021	,075	,369**	,139	,176*	-,117	,241*	-,028	,115	-,206
	Sig. (2-tailed)	,658	,261	,669	,136	,218	,858	,410	,005	,219	,046	,377	,034	,791	,403	,159
RNFL_Mean_T1_[µm]_1	Pearson Correlation	,215*	,133	,303*	,110	,176	,103	-,018	,124	-,162	,180*	-,008	,139	,034	,186	-,117
	Sig. (2-tailed)	,041	,328	,036	,216	,200	,370	,847	,366	,152	,042	,955	,224	,751	,178	,427

RNFL_Mean _T2_[μm]_1	Pearson Correlation	,104	,234	,417**	,006	-,084	-,107	-,104	,376**	-,074	,150	-,055	,178	,113	,050	-,234
	Sig. (2-tailed)	,331	,085	,004	,948	,548	,356	,254	,005	,515	,094	,687	,121	,287	,724	,113
RNFL_Mean _I1_[μm]_1	Pearson Correlation	,122	,033	,119	,065	-,089	,006	,076	-,007	,061	,145	,250	,057	-,146	-,164	,161
	Sig. (2-tailed)	,249	,805	,421	,467	,513	,956	,401	,962	,592	,103	,057	,619	,163	,232	,274
RNFL_Mean _I2_[μm]_1	Pearson Correlation	,038	,038	,066	,118	-,024	,034	,145	,364**	,079	,156	,139	,134	-,040	-,026	-,035
	Sig. (2-tailed)	,718	,778	,655	,182	,859	,767	,110	,006	,485	,079	,293	,241	,700	,850	,814
RNFL_Mean _C0_[μm]_2	Pearson Correlation	-,104	-,109	-,016	,176	,079	,053	-,344**	-,346**	-,175	,019	-,055	-,043	-,134	-,054	,423*
	Sig. (2-tailed)	,426	,425	,936	,157	,565	,790	,006	,010	,354	,880	,679	,829	,298	,699	,025
RNFL_Mean _N1_[μm]_2	Pearson Correlation	,173	,172	-,380*	,125	,064	,394*	,212	,131	,298	-,044	,135	,229	-,084	-,057	,025
	Sig. (2-tailed)	,183	,204	,046	,316	,641	,038	,096	,341	,110	,730	,312	,242	,516	,681	,900
RNFL_Mean _N2_[μm]_2	Pearson Correlation	,141	,179	-,057	,029	-,043	-,035	,026	,170	,224	,039	,011	,293	-,016	,038	-,679**

	Sig. (2-tailed)	,278	,186	,773	,814	,756	,861	,840	,214	,233	,759	,935	,130	,899	,785	,000
RNFL_Mean _S1_[µm]_2	Pearson Correlation	,379**	,393**	,189	,015	-,290*	-,420*	,256*	,269*	,312	,176	,191	-,029	-,148	-,159	-,292
	Sig. (2-tailed)	,003	,003	,336	,902	,032	,026	,043	,047	,093	,160	,151	,882	,252	,251	,132
RNFL_Mean _S2_[µm]_2	Pearson Correlation	,293*	,303*	,009	-,161	-,228	-,257	,266*	,366**	,301	,043	-,033	-,017	-,028	,001	-,479**
	Sig. (2-tailed)	,022	,023	,964	,197	,094	,186	,035	,006	,106	,734	,805	,930	,831	,994	,010
RNFL_Mean _T1_[µm]_2	Pearson Correlation	,107	,101	,147	,056	,036	,129	,298*	,150	,107	,043	,087	-,073	-,069	,029	-,354
	Sig. (2-tailed)	,410	,461	,455	,656	,794	,514	,018	,274	,573	,736	,517	,712	,595	,833	,064
RNFL_Mean _T2_[µm]_2	Pearson Correlation	,215	,172	,236	,115	-,034	,090	,299*	,158	-,418*	,232	,210	,487**	-,276*	-,086	-,663**
	Sig. (2-tailed)	,100	,208	,236	,366	,804	,654	,018	,253	,024	,067	,121	,010	,031	,539	,000
RNFL_Mean _I1_[µm]_2	Pearson Correlation	-,004	-,052	-,363	,046	,034	,517**	,129	,002	,000	,073	,211	,287	-,110	-,110	-,199
	Sig. (2-tailed)	,977	,705	,057	,712	,804	,005	,314	,987	1,000	,563	,111	,138	,395	,430	,311
RNFL_Mean	Pearson Correlation	,140	,109	,176	-,076	,031	,134	,129	,358**	-,191	,249*	,143	,348	-,107	-,029	-,695**

_I2_[µm]_2																
	Sig. (2-tailed)	,282	,426	,370	,544	,824	,495	,312	,007	,313	,045	,285	,070	,407	,833	,000
RNFL_Mean _C0_[µm]_3	Pearson Correlation	-,068	-,084	-,309*	,121	,019	-,009	,001	-,438*	-,055	,003	-,173	,100	,164	,105	,426**
	Sig. (2-tailed)	,645	,688	,037	,280	,934	,940	,994	,041	,633	,977	,429	,385	,260	,634	,003
RNFL_Mean _N1_[µm]_3	Pearson Correlation	,099	-,044	,168	,116	-,152	,023	-,099	,049	-,272*	,092	,079	,020	-,070	,008	-,069
	Sig. (2-tailed)	,503	,836	,263	,303	,501	,838	,382	,828	,016	,417	,720	,859	,631	,971	,649
RNFL_Mean _N2_[µm]_3	Pearson Correlation	-,105	,292	-,106	-,106	-,375	,033	,010	,074	,026	,114	-,078	-,071	,240	,147	,010
	Sig. (2-tailed)	,476	,157	,482	,347	,086	,775	,927	,743	,824	,314	,722	,538	,097	,503	,945
RNFL_Mean _S1_[µm]_3	Pearson Correlation	-,068	-,058	-,045	-,017	-,571**	-,068	,001	-,030	-,105	,288**	,169	,167	,063	-,110	,144
	Sig. (2-tailed)	,648	,783	,769	,878	,005	,553	,992	,895	,359	,010	,441	,143	,668	,617	,339
RNFL_Mean _S2_[µm]_3	Pearson Correlation	,104	,277	,047	-,129	-,415	-,022	,078	,338	-,071	,337**	,237	,146	-,255	-,445*	-,157
	Sig. (2-tailed)	,482	,179	,754	,249	,055	,846	,489	,124	,539	,002	,277	,202	,078	,033	,299

RNFL_Mean _T1_[µm]_3	Pearson Correlation	,211	,136	,290	,123	-,004	-,053	-,065	-,185	-,333**	,283*	-,114	,217	-,283*	-,023	-,204
	Sig. (2-tailed)	,149	,516	,051	,272	,986	,643	,565	,410	,003	,011	,604	,057	,049	,918	,174
RNFL_Mean _T2_[µm]_3	Pearson Correlation	,114	,104	,197	,055	-,165	-,116	,031	,506*	-,076	,231*	-,006	,197	-,309*	-,416*	-,197
	Sig. (2-tailed)	,439	,622	,189	,627	,463	,313	,785	,016	,510	,039	,979	,084	,031	,048	,189
RNFL_Mean _I1_[µm]_3	Pearson Correlation	,059	,072	,085	,058	-,160	-,046	-,131	-,323	-,178	,129	-,021	,091	,156	,222	-,076
	Sig. (2-tailed)	,689	,733	,572	,605	,478	,692	,246	,143	,118	,253	,923	,430	,284	,308	,617
RNFL_Mean _I2_[µm]_3	Pearson Correlation	,028	,308	-,018	,047	-,059	-,030	,205	,557**	-,128	,117	,209	,066	,054	-,244	-,090
	Sig. (2-tailed)	,848	,134	,903	,679	,793	,796	,068	,007	,263	,301	,337	,567	,711	,263	,553
GCL_Mean_ C0_µm_1	Pearson Correlation	,031	,024	-,078	,228**	,251	,099	-,075	-,008	-,051	-,136	-,036	-,114	,083	-,069	,426**
	Sig. (2-tailed)	,766	,861	,597	,009	,062	,387	,411	,954	,654	,125	,784	,322	,427	,617	,003
GCL_Mean_ N1_[µm]_1	Pearson Correlation	-,012	-,035	,312*	-,162	,041	-,090	-,048	,016	-,012	,214*	-,120	,029	-,117	,126	,138

	Sig. (2-tailed)	,909	,794	,031	,066	,762	,435	,597	,907	,915	,015	,367	,799	,265	,358	,350
GCL_Mean_ N2_[µm]_1	Pearson Correlation	,035	,122	-,109	-,243**	-,085	-,104	,126	-,021	-,075	-,041	-,138	-,153	-,056	-,042	,096
	Sig. (2-tailed)	,742	,366	,462	,006	,533	,363	,164	,879	,511	,644	,296	,182	,597	,763	,517
GCL_"Mean _S1_[µm]_1	Pearson Correlation	,246*	,233	,168	-,299**	-,340*	-,115	,082	-,021	-,098	,154	-,010	-,066	-,189	-,107	,052
	Sig. (2-tailed)	,018	,081	,254	,001	,010	,316	,367	,878	,387	,084	,938	,567	,070	,436	,727
GCL_"Mean _S2_[µm]_1	Pearson Correlation	-,047	-,032	-,125	-,132	-,094	-,158	,051	-,240	-,010	,074	-,071	-,077	-,164	,057	,173
	Sig. (2-tailed)	,654	,812	,399	,135	,490	,168	,573	,075	,927	,409	,594	,502	,116	,677	,239
GCL_Mean_ T1_[µm]_1	Pearson Correlation	,116	,105	,127	-,236**	-,149	-,041	,119	,119	-,022	-,038	-,119	-,151	-,073	,054	,144
	Sig. (2-tailed)	,272	,442	,390	,007	,278	,723	,193	,387	,849	,668	,372	,186	,490	,696	,328
GCL_Mean_ T2_[µm]_1	Pearson Correlation	,015	,063	,009	-,189*	-,238	-,189	,102	,203	-,029	,246**	-,014	,196	-,147	,059	-,086
	Sig. (2-tailed)	,891	,645	,953	,034	,083	,099	,266	,141	,801	,006	,920	,087	,165	,672	,567
GCL_Mean_	Pearson Correlation	,133	,118	-,045	-,227**	-,190	-,062	,050	-,171	,040	,091	,061	-,157	-,214*	-,130	,163

I1_[μm]_1																
	Sig. (2-tailed)	,207	,384	,764	,010	,161	,588	,580	,209	,724	,309	,646	,169	,039	,344	,267
GCL_Mean_ I2_[μm]_1	Pearson Correlation	-,062	,054	-,008	-,094	-,047	-,077	,038	,178	-,116	,020	-,247	,029	,045	,142	0,000
	Sig. (2-tailed)	,558	,692	,956	,289	,734	,501	,674	,189	,305	,821	,059	,799	,667	,300	1,000
GCL_Mean_ C0_[μm]_2	Pearson Correlation	-,257*	-,275*	-,050	,072	,110	,126	-,370**	-,286*	-,085	-,089	-,128	-,076	,055	,031	,574**
	Sig. (2-tailed)	,046	,042	,802	,567	,426	,523	,003	,034	,654	,479	,342	,702	,671	,827	,001
GCL_Mean_ N1_[μm]_2	Pearson Correlation	,299*	,235	-,250	-,063	,147	,196	-,009	,134	,081	-,084	-,042	,332	-,060	,010	,076
	Sig. (2-tailed)	,019	,084	,200	,618	,285	,318	,943	,328	,670	,507	,755	,084	,645	,943	,702
GCL_Mean_ N2_[μm]_2	Pearson Correlation	,010	-,066	-,368	,059	-,022	,115	,169	,073	,150	-,165	-,186	-,329	-,058	-,013	,225
	Sig. (2-tailed)	,941	,632	,054	,642	,874	,562	,185	,595	,428	,190	,165	,088	,658	,926	,250
GCL_Mean_ S1_[μm]_2	Pearson Correlation	,080	,020	-,171	-,047	-,211	-,178	,109	,142	,035	-,132	-,167	-,225	-,093	,007	-,085
	Sig. (2-tailed)	,539	,883	,385	,708	,122	,364	,395	,302	,855	,293	,215	,250	,476	,961	,668

GCL_Mean_ S2_[µm]_2	Pearson Correlation	-,097	-,163	-,599**	-,013	,040	,354	,037	-,120	,035	-,178	-,204	-,117	,003	,134	-,006
	Sig. (2-tailed)	,459	,234	,001	,919	,771	,064	,773	,384	,854	,155	,128	,555	,981	,338	,976
GCL_Mean_ T1_[µm]_2	Pearson Correlation	,087	,017	-,230	-,006	,077	,072	-,184	,003	,038	-,095	-,196	-,276	-,023	,034	,065
	Sig. (2-tailed)	,506	,903	,238	,964	,576	,714	,149	,985	,840	,450	,144	,155	,860	,806	,743
GCL_Mean_ T2_[µm]_2	Pearson Correlation	,259*	,227	-,550**	-,141	-,209	,122	,198	,197	,288	-,064	,090	-,096	-,070	-,152	-,205
	Sig. (2-tailed)	,046	,100	,003	,269	,129	,546	,123	,154	,130	,616	,514	,635	,593	,282	,304
GCL_Mean_ I1_[µm]_2	Pearson Correlation	,122	,052	-,315	,016	,075	,020	-,089	-,002	,104	-,058	-,105	,036	-,119	-,024	-,179
	Sig. (2-tailed)	,348	,708	,103	,900	,588	,919	,486	,989	,583	,645	,435	,855	,363	,867	,362
GCL_Mean_ I2_[µm]_2	Pearson Correlation	,190	,158	-,112	-,090	-,184	-,236	,133	,219	,078	-,020	-,038	-,144	-,161	-,078	-,065
	Sig. (2-tailed)	,143	,250	,570	,475	,179	,226	,300	,108	,682	,875	,779	,466	,215	,581	,743
GCL_Mean_ C0_[µm]_3	Pearson Correlation	-,066	-,188	-,264	,053	-,122	,077	,142	-,123	-,048	-,043	,096	,076	,043	-,444*	,398**

	Sig. (2-tailed)	,655	,368	,077	,638	,587	,505	,210	,585	,673	,708	,663	,508	,767	,034	,006
GCL_Mean_ N1_[μm]_3	Pearson Correlation	-,152	-,084	-,120	-,169	,173	,069	,006	-,342	-,010	-,099	-,119	-,126	-,074	,334	,061
	Sig. (2-tailed)	,302	,690	,426	,131	,441	,549	,960	,119	,928	,384	,590	,270	,615	,119	,689
GCL_Mean_ N2_[μm]_3	Pearson Correlation	-,127	-,527**	-,014	-,078	,025	-,059	,080	-,022	-,034	-,019	-,355	-,058	-,081	,118	,118
	Sig. (2-tailed)	,391	,007	,926	,490	,913	,607	,478	,922	,766	,867	,096	,615	,580	,592	,433
GCL_Mean_ S1_[μm]_3	Pearson Correlation	-,204	-,012	-,233	-,170	-,113	,090	,054	-,303	-,070	,046	-,109	-,015	-,076	,290	-,027
	Sig. (2-tailed)	,165	,955	,120	,129	,617	,435	,634	,170	,544	,685	,619	,894	,602	,179	,860
GCL_Mean_ S2_[μm]_3	Pearson Correlation	,000	-,078	,057	-,102	,007	-,107	-,023	-,508*	-,060	,104	-,318	,029	-,255	,238	-,059
	Sig. (2-tailed)	,998	,710	,706	,366	,976	,350	,836	,016	,600	,361	,139	,799	,077	,275	,696
GCL_Mean_ T1_[μm]_3	Pearson Correlation	-,189	-,264	-,204	-,170	-,058	,033	,049	,089	-,034	,002	-,155	-,090	,056	,283	,119
	Sig. (2-tailed)	,199	,202	,174	,129	,796	,773	,665	,694	,767	,984	,480	,435	,703	,191	,430
GCL_Mean_	Pearson Correlation	-,148	-,177	-,162	-,226*	-,374	-,125	-,061	-,127	-,151	,103	-,032	,011	-,117	-,011	-,049

T2_[μm]_3	Sig. (2-tailed)	,316	,398	,281	,042	,087	,275	,589	,574	,186	,363	,886	,925	,425	,960	,748
GCL_Mean_I1_[μm]_3	Pearson Correlation	-,196	-,146	-,201	-,172	-,132	,063	,012	-,265	,057	-,075	-,124	-,146	,046	,341	,062
	Sig. (2-tailed)	,183	,487	,180	,125	,557	,586	,917	,234	,619	,507	,574	,202	,754	,112	,681
GCL_Mean_I2_[μm]_3	Pearson Correlation	-,310*	-,448*	-,190	,060	,176	,005	-,014	-,422	,019	-,060	-,322	-,087	,036	,241	,163
	Sig. (2-tailed)	,032	,025	,207	,593	,435	,966	,904	,050	,869	,595	,134	,449	,804	,268	,280
IPL_Mean_C0_μm_1	Pearson Correlation	,028	,041	-,068	,190*	,017	-,092	-,133	-,055	-,002	-,150	-,016	-,179	,090	-,097	,397**
	Sig. (2-tailed)	,789	,761	,644	,031	,900	,421	,142	,690	,986	,091	,905	,116	,391	,479	,005
IPL_Mean_N1_[μm]_1	Pearson Correlation	-,074	-,022	,125	-,184*	-,047	-,077	-,045	,096	-,015	,172	-,118	,024	-,131	,063	,173
	Sig. (2-tailed)	,484	,870	,399	,037	,729	,504	,621	,482	,898	,053	,372	,832	,211	,649	,238
IPL_Mean_N2_[μm]1	Pearson Correlation	,009	,094	-,182	-,200*	-,105	-,058	,081	-,062	-,059	-,028	-,175	-,153	-,005	-,035	,070
	Sig. (2-tailed)	,934	,485	,216	,023	,439	,616	,372	,648	,601	,754	,184	,182	,961	,800	,634

IPL_Mean_S 1_[µm]_1	Pearson Correlation	,200	,308*	,005	-,259**	-,292*	-,148	,065	,090	-,121	,161	,042	,019	-,214*	-,062	0,000
	Sig. (2-tailed)	,056	,020	,975	,003	,029	,197	,475	,512	,285	,069	,751	,868	,040	,651	1,000
IPL_Mean_S 2_[µm]_1	Pearson Correlation	-,121	-,049	-,232	-,125	-,049	-,168	,011	-,250	-,079	,093	-,121	-,020	-,151	,040	,131
	Sig. (2-tailed)	,249	,718	,113	,159	,720	,141	,907	,063	,487	,295	,361	,861	,148	,770	,376
IPL_Mean_T 1_[µm]_1	Pearson Correlation	,156	,209	,049	-,199*	-,295*	-,053	,092	,183	-,031	,046	,032	-,065	-,135	-,112	,112
	Sig. (2-tailed)	,139	,122	,738	,024	,029	,647	,311	,180	,784	,607	,813	,571	,198	,421	,448
IPL_Mean_T 2_[µm]_1	Pearson Correlation	-,075	,096	-,108	-,171	-,214	-,216	-,014	,123	-,152	,284**	-,053	,211	-,097	,011	-,118
	Sig. (2-tailed)	,482	,486	,469	,056	,121	,059	,876	,377	,182	,001	,700	,065	,360	,937	,430
IPL_Mean_I 1_[µm]_1	Pearson Correlation	,085	,123	,020	-,223*	-,282*	,008	,088	-,065	,012	,125	,160	-,023	-,283**	-,121	,018
	Sig. (2-tailed)	,421	,364	,895	,011	,035	,945	,333	,636	,913	,161	,227	,844	,006	,378	,904
IPL_Mean_I 2_[µm]_1	Pearson Correlation	-,087	,054	-,082	-,119	-,077	-,026	,003	,108	-,104	,075	-,201	,003	,031	,094	,007

	Sig. (2-tailed)	,408	,692	,580	,178	,575	,823	,971	,429	,360	,401	,127	,980	,768	,494	,961
IPL_Mean_C 0_[µm]_2	Pearson Correlation	-,262*	-,261	-,138	-,069	-,053	,215	-,238	-,283*	-,019	,035	,082	,129	,052	-,109	,095
	Sig. (2-tailed)	,041	,050	,502	,586	,695	,292	,060	,033	,923	,781	,535	,532	,692	,428	,643
IPL_Mean_N 1_[µm]_2	Pearson Correlation	,233	,176	-,231	,032	,051	,228	,054	,056	,118	-,002	,010	-,078	-,197	-,105	,407*
	Sig. (2-tailed)	,071	,190	,256	,803	,704	,262	,675	,680	,548	,987	,940	,707	,128	,444	,039
IPL_Mean_N 2_[µm]_2	Pearson Correlation	,008	-,048	-,317	,284*	,146	,022	,144	,332*	,052	-,131	-,196	-,005	-,021	,151	,024
	Sig. (2-tailed)	,949	,723	,115	,022	,279	,916	,259	,012	,792	,298	,137	,981	,872	,271	,906
IPL_Mean_S 1_[µm]_2	Pearson Correlation	,214	,133	-,200	,121	,000	,017	,089	,120	-,074	-,003	-,012	-,267	-,188	-,082	,248
	Sig. (2-tailed)	,098	,325	,328	,337	,997	,934	,489	,373	,707	,982	,928	,188	,146	,552	,222
IPL_Mean_S 2_[µm]_2	Pearson Correlation	-,114	-,161	-,499**	,150	,123	,047	,049	,130	,094	-,159	-,234	,097	,067	,228	-,121
	Sig. (2-tailed)	,383	,232	,009	,232	,364	,821	,702	,335	,633	,206	,074	,638	,605	,094	,557
IPL_Mean_T	Pearson Correlation	-,009	-,079	-,126	,089	-,009	,089	-,042	-,033	-,157	,028	,007	-,177	-,178	-,083	,122

1_[µm]_2																
	Sig. (2-tailed)	,943	,561	,539	,480	,948	,664	,742	,807	,426	,823	,956	,387	,169	,545	,553
IPL_Mean_T 2_[µm]_2	Pearson Correlation	,135	,099	-,545**	-,108	-,125	,126	,201	,094	,074	,080	,177	,231	-,166	-,186	-,350
	Sig. (2-tailed)	,303	,466	,005	,398	,360	,549	,116	,489	,714	,535	,189	,266	,205	,179	,086
IPL_Mean_I 1_[µm]_2	Pearson Correlation	,090	,004	-,114	,182	,048	,149	-,018	-,012	-,040	-,018	-,073	-,053	-,184	-,047	,089
	Sig. (2-tailed)	,490	,976	,580	,146	,725	,469	,887	,932	,840	,886	,582	,796	,156	,733	,664
IPL_Mean_I 2_[µm]_2	Pearson Correlation	,197	,152	-,029	,161	,039	-,153	,028	,241	-,032	-,147	-,230	-,242	-,063	,145	,131
	Sig. (2-tailed)	,127	,259	,887	,200	,774	,455	,828	,070	,872	,242	,080	,233	,632	,290	,523
IPL_Mean_C 0_[µm]_3	Pearson Correlation	-,064	-,511*	-,108	,159	,041	,068	,057	-,234	-,043	-,033	-,226	,106	,160	-,207	,276
	Sig. (2-tailed)	,665	,013	,467	,157	,864	,551	,613	,321	,708	,773	,324	,351	,273	,367	,057
IPL_Mean_N 1_[µm]_3	Pearson Correlation	-,257	-,598**	-,096	-,021	,211	,073	,105	,137	,051	-,123	-,373	-,140	,048	,181	,124
	Sig. (2-tailed)	,078	,003	,515	,852	,372	,518	,354	,564	,653	,276	,096	,215	,742	,432	,401

IPL_Mean_N 2_[μm]_3	Pearson Correlation	-,266	-,651**	-,076	,029	,269	-,014	,111	-,036	-,057	,038	-,407	-,040	-,090	,147	,104
	Sig. (2-tailed)	,067	,001	,608	,794	,251	,901	,327	,882	,614	,739	,067	,723	,537	,525	,480
IPL_Mean_S 1_[μm]_3	Pearson Correlation	-,314*	-,707**	-,111	-,032	,177	,022	-,008	-,141	-,112	,042	-,430	,043	-,099	,333	-,047
	Sig. (2-tailed)	,030	,000	,455	,777	,454	,850	,946	,552	,323	,714	,052	,704	,496	,140	,751
IPL_Mean_S 2_[μm]_3	Pearson Correlation	-,207	-,483*	-,012	-,018	,184	-,129	,050	-,403	-,083	,040	-,608**	-,031	-,211	,242	0,000
	Sig. (2-tailed)	,158	,020	,933	,873	,438	,256	,662	,078	,465	,724	,003	,785	,146	,291	1,000
IPL_Mean_T 1_[μm]_3	Pearson Correlation	-,330*	-,668**	-,168	-,110	,182	,021	,101	-,011	,012	-,054	-,350	-,096	,098	,447*	,191
	Sig. (2-tailed)	,022	,000	,253	,327	,443	,851	,371	,963	,919	,636	,120	,395	,502	,042	,194
IPL_Mean_T 2_[μm]_3	Pearson Correlation	-,424**	-,584**	-,273	-,170	,219	-,074	-,022	-,153	-,082	,159	-,468*	,019	-,068	,327	,030
	Sig. (2-tailed)	,003	,003	,060	,130	,354	,516	,847	,519	,468	,159	,033	,866	,641	,148	,840
IPL_Mean_I 1_[μm]_3	Pearson Correlation	-,381**	-,740**	-,178	-,071	,124	,102	,061	,021	-,001	-,034	-,441*	-,131	,055	,281	,171

	Sig. (2-tailed)	,007	,000	,227	,532	,601	,367	,591	,931	,991	,763	,045	,248	,706	,217	,245
IPL_Mean_I 2_[μm]_3	Pearson Correlation	-,294*	-,626**	-,117	,082	,158	-,021	,045	-,190	-,004	,008	-,418	-,088	,045	,152	,234
	Sig. (2-tailed)	,042	,001	,430	,464	,507	,852	,689	,423	,969	,942	,060	,437	,761	,511	,109

Note: **. Correlation is significant at the 0.01 level (2-tailed).
***. Correlation is significant at the 0.05 level (2-tailed).**

Supplementary Table s2. Availability and handling of potential confounding variables.

Potential confounding variable	Availability in the present study	Handling in the analysis/manuscript
Age and Sex	Available	Reported at baseline and compared between groups.
Visual acuity (VA) and Intraocular pressure (IOP)	Available	Reported in the clinical/functional results and included among the analyzed functional/clinical variables.
Retinal disease	Assessed	Controlled through exclusion criteria.
Optic nerve abnormalities	Assessed	Controlled through exclusion criteria.
Neurological disease	Assessed	Controlled through exclusion criteria.
Major chronic illnesses	Assessed	Controlled through exclusion criteria, including systemic diseases potentially affecting retinal structure.
High refractive error	Assessed	Controlled through exclusion criteria.
OCT image quality	Available/controlled	Controlled through a predefined minimum signal-quality threshold and manual review of segmentation; scans with relevant artifacts or persistent segmentation errors were excluded.
Tobacco use	Not available	Not formally adjusted; acknowledged as a potential residual confounder.
Migraine	Not available	Not formally adjusted; acknowledged as a potential residual confounder.
Vaccination status	Not available/incomplete	Not formally adjusted; acknowledged as a potential residual confounder.
Number of SARS-CoV-2 infections	Not available/incomplete	Not formally adjusted; acknowledged as a potential residual confounder.
Severity of acute COVID-19 episode	Not available/incomplete	Not formally adjusted; acknowledged as a potential residual confounder.
Hospitalization during acute COVID-19	Not available/incomplete	Not formally adjusted; acknowledged as a potential residual confounder.
Body mass index	Not available	Not formally adjusted; acknowledged as a potential residual confounder.
Axial length	Not available	Not formally adjusted; acknowledged as a potential residual confounder.
Refractive-error distribution within the allowed range	Partially controlled	Extreme refractive errors were excluded; refractive error was not adjusted as a continuous covariate.

Note: This table summarizes the availability and handling of selected potential confounding variables in the present study. Variables classified as “available” or “assessed” were reported, compared between groups, or controlled through inclusion/exclusion criteria and OCT quality-control procedures. Variables classified as “not available” or “not available/incomplete” were not formally adjusted for in the longitudinal analyses and are acknowledged as potential residual confounders.
